# Supplementary material for: A Co-Culture-Based Multiparametric Imaging Technique to Dissect Local H2O2 Signals with Targeted HyPer7
Source: Biosensors (Basel). 2021 Sep 14;11(9):338. doi: 10.3390/bios11090338 (PMC8466187; doi:10.3390/bios11090338)
Supplement: Supplementary file 1 [file biosensors-11-00338-s001.zip › biosensors-1374732-SI.pdf]

biosensors

MDPI

*Supplementary Materials*

# A co-culture-based multiparametric imaging technique to dissect local $H_2O_2$ signals with targeted HyPer7

**Melike Secilmis[1,#], Hamza Y. Altun[1,#], Johannes Pilic[2], Yusuf C. Erdogan[1,2], Zeynep Cokluk[1], Busra N. Ata[1], Gulsah Sevimli[1], Asal Ghaffari Zaki[1], Esra N. Yigit[3,4], Gürkan Öztürk[3,5], Roland Malli[2,6,*] & Emrah Eroglu[1,2,3,7*]**

[1] Molecular Biology, Genetics and Bioengineering Program, Sabanci University, Istanbul, Turkey
[2] Molecular Biology and Biochemistry, Gottfried Schatz Research Center, Medical University of Graz, Austria
[3] Research Institute for Health Sciences and Technologies (SABITA), Istanbul Medipol University, Istanbul, Turkey
[4] Department of Biotechnology, Gebze Technical University, Kocaeli, Turkey
[5] Physiology Department, International School of Medicine, Istanbul Medipol University, Istanbul, Turkey
[6] BioTechMed Graz, Mozartgasse 12/II, 8010 Graz, Austria
[7] Nanotechnology Research and Application Center, Sabanci University Istanbul, Turkey
[#] These authors contributed equally
* Correspondence: roland.malli@medunigraz.at or emrah.eroglu@sabanciuniv.edu

## Supplementary Information

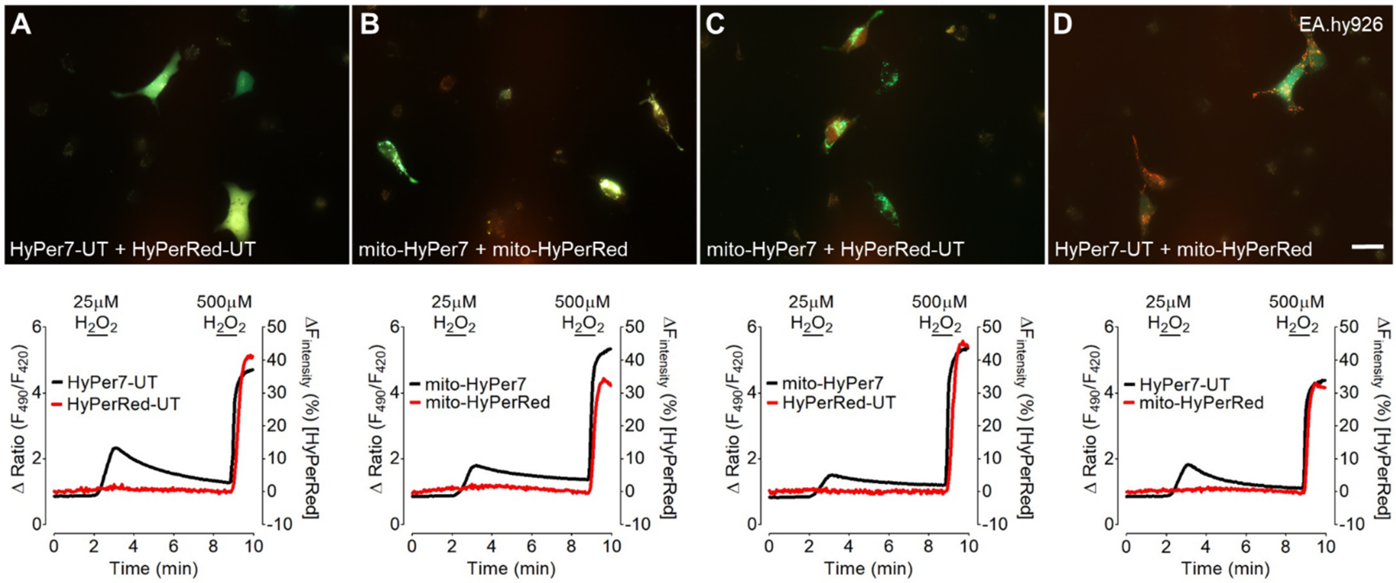


**Figure S1**. Multispectral imaging of differentially targeted HyPer7 and HyPerRed in transiently transfected EA.hy926 cells. Upper panel shows representative widefield fluorescence images of transiently transfected EA.hy926 cells co-expressing differently targeted HyPer7 and HyPerRed and lower panels show respective representative real-time traces of HyPer7 signals in black, and HyPerRed signals in red in response to constitutive administration of 25 µM and 500 µM exogenous $H_2O_2$. (A) HyPer7-UT and HyPerRed-UT (n=3/9), (B) mito-HyPer7 and mito-HyPerRed (n=3/17), (C) mito-HyPer7 and HyPerRed-UT (n=3/9), (D) HyPer7-UT and mito-HyPerRed (n=3/14). Scale bar represents 20 µm.

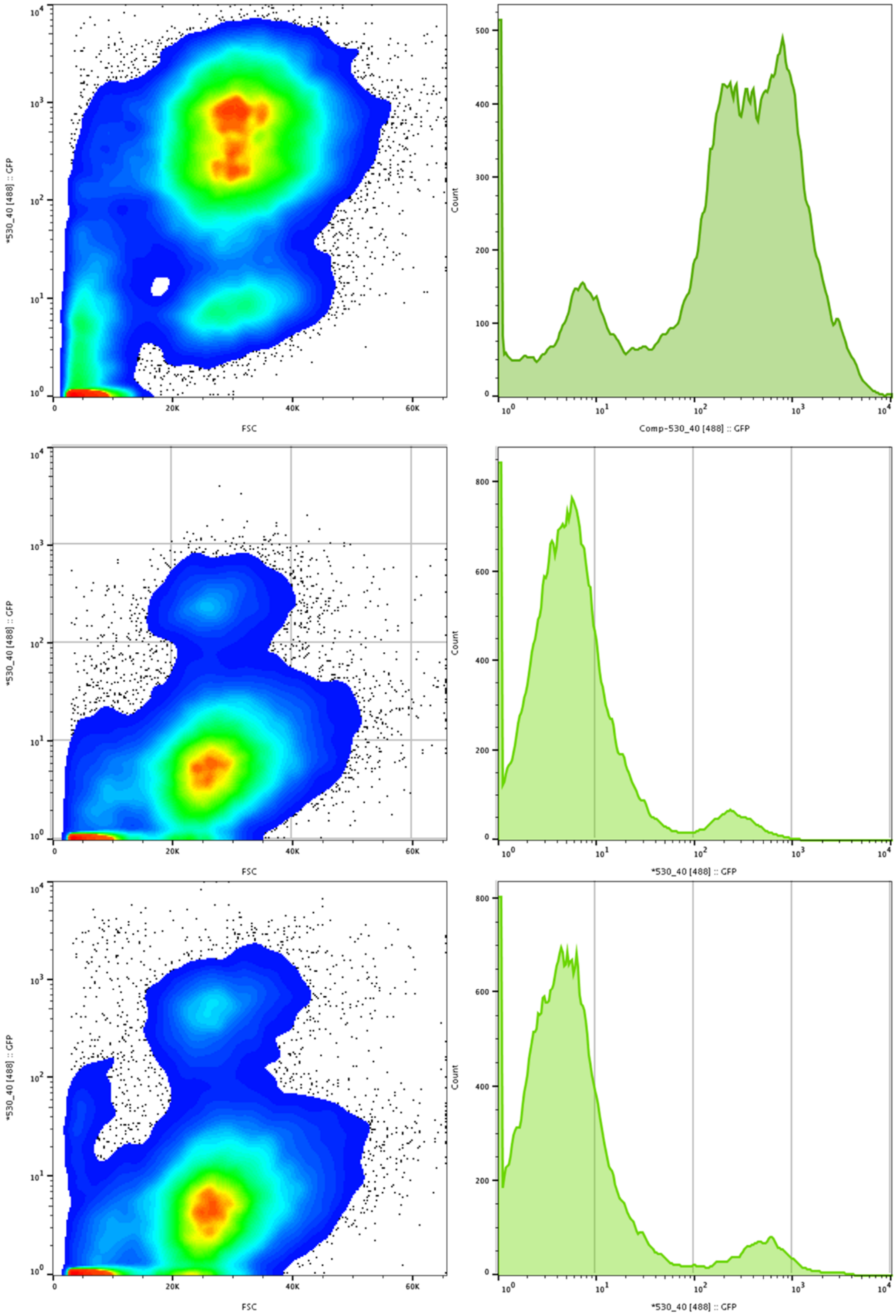


**Figure S2**. Fluorescence assisted cell sorting results of positively transduced EA.hy926 cells expressing the nuclear targeted HyPer7 (upper panels), cytosolic targeted HyPer7 (middle panels) and mitochondria targeted HyPer7 (lower panels). Left panels show scatter dot plots of GFP intensities of cells (y-axis) against forward scattering (x-axis). Right panels show histograms of each cell population indicating number of cells (y-axis) against GFP fluorescence intensities (x-axis). Upper 30% of GFP positive cells have been gated.

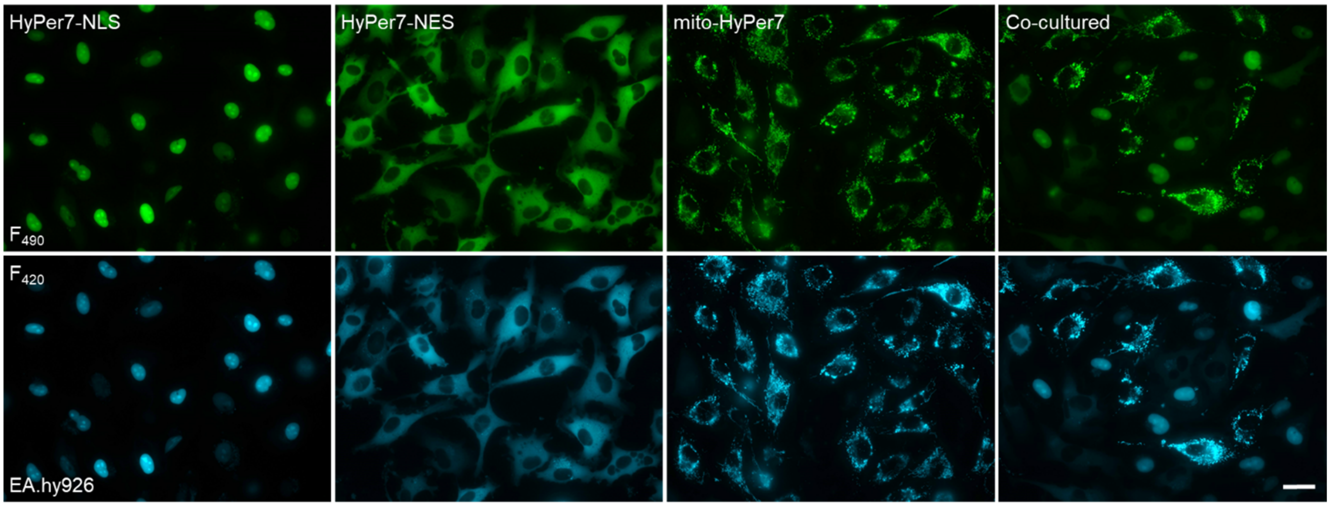


**Figure S3.** EA.hy926 cells stably expressing differentially targeted HyPer7 biosensors. Representative widefield fluorescence images of EA.hy926 cells expressing nuclear-, cytosolic-, and mitochondria-targeted HyPer7 either separately (first three panels from the left as indicated) or in mixed co-culture (very right panel). Upper panels show representative images in the HyPer high channel (Ex: 490 nm, Em: 520 nm), and lower panels show representative images captured in the HyPer low channel (Ex: 420 nm, Em: 520 nm).

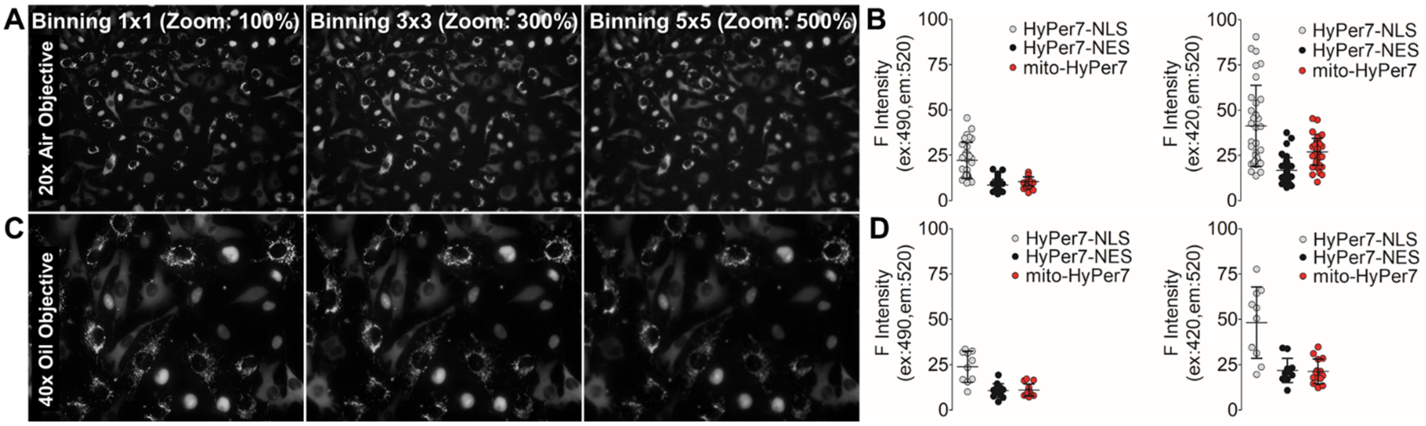


**Figure S4.** Camera settings for optimum spatial resolution of co-cultured cells. (A) Micrographs show representative widefield images of a single field of view imaged with a 20x air objective with different camera binning settings ranging from 1x1 to 5x5 as indicated above the images. (B) Scatter dot plot shows the analysis of fluorescence intensity of cells shown in panel A expressing the nuclear-targeted HyPer7 (grey dots, n=32), cytosolic-targeted HyPer7 (black dots, n=40), and mitochondria-targeted HyPer7 (red dots, n=42) in the HyPer high channel (Ex: 490 nm, Em: 520 nm, left panel) and HyPer low channel (Ex: 420 nm, Em: 520 nm, right panel). (C) This figure shows the same experimental setup shown in panels A and B, here instead imaged with a 40x oil objective. (D) Scatter dot plot shows the analysis of fluorescence intensity of cells in panel C expressing the nuclear-targeted HyPer7 (grey dots, n=10), cytosolic-targeted HyPer7 (black dots, n=12), and mitochondria-targeted HyPer7 (red dots, n=14) in the HyPer high channel (Ex: 490 nm, Em: 520 nm, left panel) and the HyPer low channel (Ex: 420 nm, Em: 520 nm, right panel). All values are given as ±SD.
